# TP53 Mutations and Survival in Osteosarcoma Patients: A Meta-Analysis of Published Data

**DOI:** 10.1155/2016/4639575

**Published:** 2016-04-27

**Authors:** Zhe Chen, Jiayi Guo, Kun Zhang, Yanxing Guo

**Affiliations:** Luoyang Orthopedic Hospital of Henan Province, Luoyang 471000, China

## Abstract

Several research groups have examined the association between TP53 mutations and prognosis in human osteosarcoma. However, the results were controversial. The purpose of this study was to evaluate the prognostic value of TP53 mutations in osteosarcoma patients. A meta-analysis was conducted with all eligible studies which quantitatively evaluated the relationship between TP53 mutations and clinical outcome of osteosarcoma patients. Eight studies with a total of 210 patients with osteosarcoma were included in this meta-analysis. The risk ratio (RR) with a 95% confidence interval (95% CI) was calculated to assess the effect of TP53 mutations on 2-year overall survival. The quantitative synthesis of 8 published studies showed that TP53 mutations were associated with 2-year overall survival in osteosarcoma patients. These data suggested that TP53 mutations had an unfavorable impact on 2-year overall survival when compared to the counterparts with wild type (WT) TP53 (RR: 1.79; 95% CI: 1.12 to 2.84; *P* = 0.01; *I* ^2^ = 0%). There was no between-study heterogeneity. TP53 mutations are an effective prognostic marker for survival of patients with osteosarcoma. However, further large-scale prospective trials should be performed to clarify the prognostic value of TP53 mutations on 3- or 5-year survival in osteosarcoma patients.

## 1. Introduction 

Osteosarcoma is the most common malignancy that occurred in bone. In the past few decades, although neoadjuvant chemotherapy and surgery have made remarkable progress to reduce tumor burden, therapeutic effectiveness of conventional therapies for metastatic osteosarcoma has remained unchanged, with a low five-year survival rate of less than 20% [1, 2]. Despite the rapid development of genetics and cell biology of osteosarcoma, further improvement in survival has not been achieved owing to the lack of effective indicators that are helpful for predicting individual clinical outcome.

Tumor protein p53 (TP53), also known as p53, BCC7, LFS1, or TRP53, is located on chromosome 17p13.1 and plays an important role in tumorigenesis [3, 4]. TP53 mutations were found in most of the human tumor tissues and were the most common genetic alterations [5–8]. Mutations in p53, tumor suppressor gene, have been proved to play a vital role in cell proliferation and in the pathogenesis of osteosarcoma [9–11]. Previous studies have shown that mutations in this gene were associated with poor prognosis in human osteosarcoma [12, 13]. In a recent study, whole-genome sequencing of tumors from 32 osteosarcoma patients showed that cancer-specific TP53 rearrangements were found in more than 50% of patients [14]. However, the clinical significance of TP53 mutations in osteosarcoma is controversial. In some reports, TP53 alterations are associated with poor response to chemotherapy and decreased survival in human osteosarcoma [9, 15–20], whereas other data showed no correlation with chemotherapy response or clinical outcomes of patients with osteosarcoma [20–22]. Therefore, it would be valuable to conduct a quantitative synthesis using rigorous methods. In this study, we conducted an updated meta-analysis of all available studies to identify whether TP53 mutations were involved in the process of cancer as a prognostic marker in patients with osteosarcoma.

## 2. Materials and Methods

### 2.1. Selection Criteria and Search Strategy

We identified all available studies that reported the association of TP53 mutations with efficacy survival in osteosarcoma. The electronic databases PubMed, Embase, Web of Science, and CNKI were searched for all articles before August 25, 2015. Searches included the terms osteosarcoma, osteosarcomas, TP53, TP53 mutation, p53, p53 mutation, and 17p13 gene. The references cited in the identified articles were also screened to complete the search.

### 2.2. Definitions and Standardization

All studies examining the association of overall survival with TP53 mutations in osteosarcoma are eligible for our meta-analysis. We accepted the studies identifying TP53 mutations in osteosarcoma regardless of the method of detection (polymerase chain reaction single-strand conformation polymorphism analysis and direct sequencing). When the same author reported results from the same patient population in two or more publications, the most recent study or the largest one was included to avoid the same patients in more than one article. Letters to the editor, reviews, and articles published in books were excluded from this meta-analysis. Two reviewers independently determined study eligibility and disagreements were resolved by consensus.

### 2.3. Data Extraction

Two investigators extracted data independently, discussed discrepancies, and reached consensus on the following standardized data-collection forms. We extracted data on characteristics of studies and patients, measurements, and results. In each report, we recorded first author's last name, publication year, number of patients, mean age, metastatic disease, stage of osteosarcoma, chemotherapy and surgery used, and survival data. The studies without the above categories were excluded. We contacted the author of primary study to request missing information related to the meta-analysis. Disagreements were resolved by discussion until a consensus was reached.

### 2.4. Statistical Analysis

Meta-analyses were performed in Excel (Microsoft) and Review Manager software (Review Manager 5.3) according to Cochrane Handbook. RR with 95% CIs was used to estimate the association between TP53 mutations and response to chemotherapy in patients with osteosarcoma. *I* ^2^ statistics were used to determine heterogeneity of the 8 studies. A *P* value < 0.05 and/or *I* ^2^ > 50% was considered significant. A fixed effect model was applied in the absence of between-study heterogeneity, while a random effect model was used when heterogeneity was observed. Using Review Manager software, possible publication bias was estimated by funnel plots. Sensitivity analysis was also performed by omitting each study or specific studies to find potential outliers. *P* values for all comparisons were two-tailed and a *P* value < 0.05 was considered significant for all tests, except those for heterogeneity.

## 3. Results

### 3.1. Study Selection

**Figure 1:**
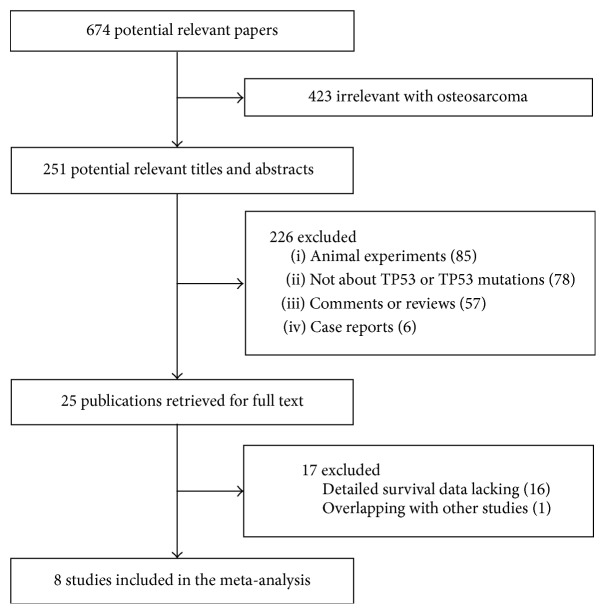
The flow chart of the included studies.

In this study, we first searched PubMed, Embase, Web of Science, and CNKI databases, and a total of 674 published articles were reviewed using the search strategy as described above. As shown in Figure 1, we initially excluded 649 publications: 423 were of other diseases; 85 were animal experiments; 78 were not for TP53; 57 were comments or reviews, and 6 were case reports. By further review of the remaining 25 literatures, 16 publications were excluded due to lack of detailed survival analysis, and 1 was excluded because of overlapping with other studies. Finally, 8 studies were included in this meta-analysis [12, 13, 23–28].

### 3.2. Characteristics of the Included Studies

The detailed characteristics of these 8 eligible studies published between 1998 and 2013 were summarized in Table 1. In total, 210 patients were included in this analysis and study sample sizes ranged from 17 to 54 with a mean of 26. Among these available studies, 4 studies were executed on osteosarcoma in high histological grades, while 1 study was executed on osteosarcoma in low or intermediate histological grades and 1 study was on osteosarcoma in various histological grades. However, grade data was not shown in 2 studies.

### 3.3. Meta-Analysis Results

**Figure 2:**
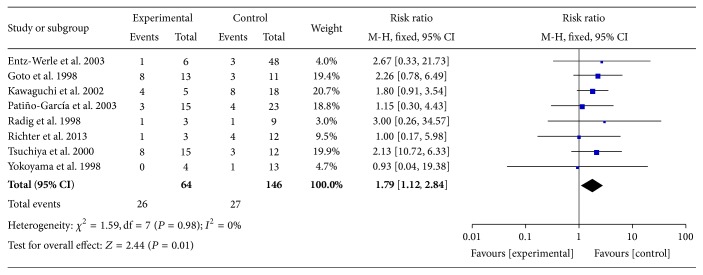
Meta-analysis (forest plot) of the 8 studies evaluating TP53 mutations in osteosarcoma for the risk of 2-year death. The name of the lead author and the RR with 95% confidence intervals are shown in each study. The total RR and 95% confidence intervals are summarized with fixed effects models.

**Figure 3:**
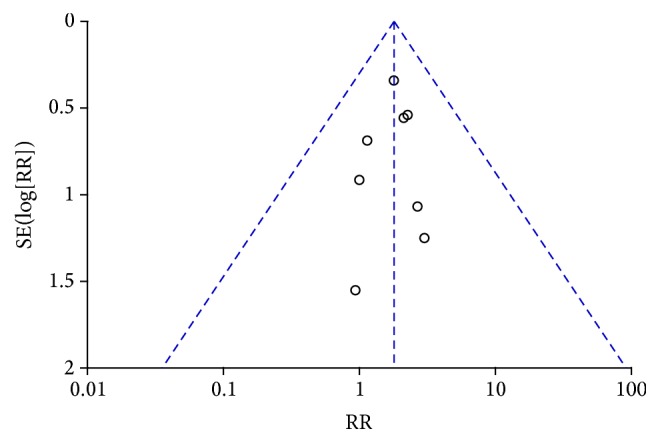
Funnel plot in the meta-analysis demonstrating that there was no obvious indication of publication bias.

There was no heterogeneity among these included studies (*I* ^2^ = 0%, Figure 2) and a fixed effects model was chosen. Meta-analysis of those 8 studies showed that TP53 mutations were remarkably associated with a higher risk of death within 2 years compared with their counterparts with WT TP53 (Figure 2, RR: 1.79; 95% CI: 1.12 to 2.84; *P* = 0.01). Sensitivity analysis showed that the pooled RR was stable and was not remarkably changed when each study was omitted (Figure 3). These analyses suggested that TP53 mutations in patients with osteosarcoma predicted poor 2-year overall survival, whereas more clinical studies should be conducted taking into account the age, sex, metastasis, histological grades, primary sites, and treatment of osteosarcoma patients.

### 3.4. Publication Bias and Sensitivity Analysis

The publication bias of the literature included in this study was assessed by means of funnel plots. The shape of the funnel plots was symmetrical, demonstrating that no publication bias existed in this analysis (Figure 3). In addition, sensitivity analysis was conducted to assess whether individual study affected final summary results. The sensitivity analysis showed that none of the studies remarkably affected the pooled RRs and CIs, and deletion of any one study had no significant effect on the final results (data not shown).

## 4. Discussion

Osteosarcoma, a malignant tumor in bone, is harmful to the health of children and adolescents, accounting for approximately 5% of tumors in childhood. But so far there have been no effective clinical prognostic markers to determine outcome of patients and response to chemotherapy. In numerous studies using sequencing, TP53 mutations have been proven to be a powerful prognostic indicator for ER-positive tumors, including breast tumors. The majority of TP53 alterations are missense mutations that occur in exons 5 to 8, highly conserved regions, and principal structural domains of the TP53 protein [11]. In the included studies, silent deletions, missense and nonsense mutations, aberrant methylation, and one single-nucleotide substitution were observed in TP53 genes of patients with osteosarcoma. TP53 mutations may promote tumorigenesis and the identification of TP53 mutations was helpful to assess the clinical features of osteosarcoma (tumor grade, type, aggressiveness, and metastatic potential) [29, 30]. A previous meta-analysis showed that TP53 status is not associated with the histologic response to chemotherapy [21], while another meta-analysis showed that high TP53 expression predicted poor overall survival and disease-free survival in patients with osteosarcoma and Ewing's sarcoma. The results obtained in these studies were conflicting. As we know, mutations may reduce the stability of proteins and induce truncated protein not detected by immunological histological chemistry. Therefore, the result of the article by Jiang and colleagues [31] is controversial. Although the other meta-analysis published in 2004 showed that TP53 gene alterations were associated with decreased survival [21], due to limitation of sample size, the conclusion was not strong. To examine the prognostic role of TP53 mutations in osteosarcoma patients, we systematically reviewed the published literature and performed a meta-analysis.

The present meta-analysis with a larger sample size showed that TP53 mutations were prognostic predictors for survival of osteosarcoma patients (RR = 1.79; 95% CI: 1.12–2.84; *P* = 0.01) (Figure 2). Sensitivity analysis suggested that the pooled RR was stable and significance of the pooled RR did not change when a single study was removed (Figure 3). In conclusion, the present meta-analysis indicated that TP53 mutations are a valuable prognostic indicator for poor prognosis in osteosarcoma patients.

However, some limitations do exist in this meta-analysis. Firstly, the sample size of this study was still small and there were only 8 available literatures with 210 osteosarcoma patients. Secondly, publication bias may exist in meta-analyses. Despite our best efforts, there still were some literatures that were not included in this meta-analysis due to the lack of detailed data. Thirdly, other factors in eligible studies may increase between-study heterogeneity. The heterogeneity may be from the individual differences of patients and medical technology. Fourthly, treatments of the patients and some osteosarcoma features (such as tumor type, aggressiveness, and metastatic potential) should be taken into account, which might be related to the survival time of patients with osteosarcoma. Therefore, further studies with larger sample sizes must be performed in the future to evaluate the prognostic significance of TP53 mutations in osteosarcoma.

In conclusion, the results from the present meta-analysis suggested that TP53 mutations are useful predictive biomarkers of 2-year overall survival in osteosarcoma patients, which will provide guidance for the clinical treatment.

## Figures and Tables

**Table 1:** Main characteristics of eligible studies.

Author (yrs)	Cases	Age (mean yrs)	HG I/II (III/IV)	Metastatic disease	Treatment	PCR exons	Deaths in 2 years, *N* (%)	Chemotherapy response (criteria)
Yokoyama et al. (1998) [23]	17	15	(8/7)	2	NC + surgery	4–8	1 (6)	6/14 (S-K)
Radig et al. (1998) [24]	18	34	10/8	0	Surgery	4–8	2 (17)	NR
Goto et al. (1998) [12]	32	16	(23/9)	8	NC + surgery	MS	14 (44)	3/31 (N90)
Tsuchiya et al. (2000) [13]	27	15	NR	2	NC + surgery	5–9	11 (41)	NR
Kawaguchi et al. (2002) [25]	23	55	(8/15)	NR	Surgery	5–9	12 (52)	NR
Patiño-García et al. (2003) [26]	41	14	NR	8	NR	5–8	7 (18)	22/41 (N90)
Entz-Werle et al. (2003) [27]	54	13	(43/11)	6	NC + surgery	MS	4 (7)	30/53 (Huvos)
Richter et al. (2013) [28]	17	34	NR	NR	Surgery	5–9	5 (31)	3/17 (Huvos)

*Note*. Exons: exons of the TP53 gene analyzed by polymerase chain reaction.

*N*: number; yrs: years; HG: histological grades; NC: neoadjuvant chemotherapy; Huvos: histological response based on the Huvos grading system; NR: not reported; N90: histological response based on >90% tumor cell necrosis; PCR: polymerase chain reaction; S-K: histological response based on Salzer-Kuntschik's classification; MS: microsatellite primers.
